# Ultrasound Sensors for Diaphragm Motion Tracking: An Application in Non-Invasive Respiratory Monitoring

**DOI:** 10.3390/s18082617

**Published:** 2018-08-09

**Authors:** Amirhossein Shahshahani, Carl Laverdiere, Sharmistha Bhadra, Zeljko Zilic

**Affiliations:** 1 Department of Electrical and Computer Engineering, McGill university, Montreal, QC H3A 0E9, Canada; sharmistha.bhadra@mcgill.ca (S.B.); zeljko.zilic@mcgill.ca (Z.Z.); 2 Faculty of Medicine, McGill University, Montreal, QC H3A 0E9, Canada; carl.laverdiere@mail.mcgill.ca

**Keywords:** non-invasive respiratory monitoring, diaphragm motion monitoring, PZT5 piezo, ultrasound, inertial sensor, pulse-oximetry sensor (PPG)

## Abstract

This paper introduces a novel respiratory detection system based on diaphragm wall motion tracking using an embedded ultrasound sensory system. We assess the utility and accuracy of this method in evaluating the function of the diaphragm and its contribution to respiratory workload. The developed system is able to monitor the diaphragm wall activity when the sensor is placed in the zone of apposition (ZOA). This system allows for direct measurements with only one ultrasound PZT5 piezo transducer. The system generates pulsed ultrasound waves at 2.2 MHz and amplifies reflected echoes. An added benefit of this system is that due to its design, the respiratory signal is less subject to motion artefacts. Promising results were obtained from six subjects performing six tests per subject with an average respiration detection sensitivity and specificity of 84% and 93%, respectively. Measurements were compared to a gold standard commercial spirometer. In this study, we also compared our measurements to other conventional methods such as inertial and photoplethysmography (PPG) sensors.

## 1. Introduction

Wearable devices providing non-invasive high resolution monitoring of living organs are essential in the hospital settings for observing physiological activity, mainly breathing and heart rate [1]. In the intensive care unit (ICU), almost all physiological parameters are measured and monitored, but the assessment of respiratory muscle is lacking [2]. Normal function of the diaphragm is critical for effective ventilation during sleep in normal subjects [3]. Monitoring of respiratory activity is needed to detect respiratory disorders, such as sleep apnoea [4], cessation of breathing in infants [5] or dyspnea. Dyspnea relates to patients having difficulty breathing, whereas apnoea refers to the cessation of airflow during sleep, preventing air from entering the lungs. Besides the respiratory rate, depth and patterns are important [6]. Central sleep apnoea (CSA) is another form of respiratory disease in which the brain temporarily fails to signal the muscles responsible for controlling respiration [7].

Numerous non-invasive devices for respiration monitoring have been proposed. Appropriate use of current monitoring systems and correct assessment of the provided data are essential in accurate diagnosis. Some direct monitoring systems are flow meters that use pressure transducer, thermal or ultrasound flow meters [8,9,10]. These sensors measure some characteristics of air (e.g., temperature, oxygen level) or the direction of airflow from nasal or oral breathing.

Aluminium nitride piezoelectric films [11,12] can measure and monitor the applied pressure on the sensor when a subject is lying on it. Physiologic activity, such as heartbeat and respiration, creates a pressure fluctuation perceived by the sensor. Many fibre-optic [13,14] and fibre-resistive [15] sensors for heart and respiratory monitoring are based on the same concept of elongation of the abdomen or chest circumference. Another example of a direct method is chest and abdomen movement analysis using accelerometer sensors [16]. Since these methods are based on the heartbeat felt on the skin and abdominal circumference changes due to breathing, they are susceptible to human motion artefact. Contact-less breathing or heart rate tracking by low frequency ultrasound sensor was reported by Arlotto et al. [17]. This system is based on detecting the time of flight of ultrasound waves. However, it is impractical, especially when the patient is dressed or covered by a blanket.

Indirect methods are based on biofeedback from organs’ activities. As an example, the photoplethysmography (PPG) sensor, as the most popular and inexpensive method for heart rate monitoring, is not a direct method for respiratory monitoring, as discussed more in the sections to follow. This sensor emits infrared light into the tissue and measures the amount of light that is scattered by the blood flow. Furthermore, this sensor measures oxygen level variation due to breathing [18,19], as well as small blood pressure variations due to the pulmonary pressure. Nonetheless, these are dependent on many parameters. For instance, the oxygen level depends on the oxygen density of the air the person is breathing [20], the function of the lungs, the latency between breaths [18], the oxygen level variation in the blood, uncertainty in breath detection during fast respirations and the depth of breathing [21]. In addition, it is not desirable to have a sensor on the finger, earlobe or forehead [22,23]. Many researchers found that the finger is the best location for PPG to extract respiratory signals [24,25,26].

ECG-based devices [27], as the most common cardio-respiratory monitoring tools, require at least two conductive electrodes being mounted on the chest skin. ECG records the electrical activity generated by heart muscle depolarizations, which propagate as electrical waves on the chest skin surface. ECG and PPG devices are the most accurate and conventional methods in hospitals for heart monitoring, especially for critically-ill patients, but not ideal for respiratory monitoring. In a study [28], the researchers described a signal processing technique that derives respiratory waveforms from ordinary ECGs, permitting reliable detection of respiratory efforts. However, the data are sensitive to motion and can cause false peaks as a breathing operation. Skin conductivity assessment (plethysmography) provides a way to measure respiratory activity by measuring the thoracic impedance changes caused by inspiration and expiration. Accordingly, this method requires at least two conductive electrodes, which makes it uncomfortable as a long-term monitoring system. Moreover, the movement-induced artefact on its signal cannot be easily removed by filtering methods [29,30].

Many of the above-mentioned conventional techniques are impractical in real-life conditions such as the patient being physically active or fully dressed. In a wearable health monitoring system, it is advantageous if the device monitors while operating with different body positions, with less constraints on hardware resources and wiring connectivities. In this paper, we report an ultrasound-based diaphragm motion tracking system to obtain a respiratory signal.

Ultrasound technology (US) is inexpensive, safe, real-time and can be used as a direct method for cardiopulmonary monitoring. Ultrasound imaging systems with an array of piezo transducers are widely used in diagnosis and imaging [31]. Zambon et al. investigated the use of point of care ultrasound to assess the function of the diaphragm [32], but it involved manual operation and was not continuous. In an earlier study by the authors, the ultrasound system based on motion tracking of the heart was employed to monitor heart and respiration rates [33]. The PZT4 piezo transducer was placed on the chest in front of the heart. In this work, the sensor is placed in the zone of apposition (ZOA) to observe only the diaphragm motions. Therefore, the system is less sensitive to upper body motion and sensor displacement than the previous work. In addition, the obtained signals of this system from six subjects are validated against a reference. The system uses one sensor, consisting of a PZT5 piezo disk transducer as a 1D tracker of the diaphragm. A mixed-signal embedded system is designed to operate in B-mode, which generates ultrasound pulses discontinuously and records ultrasound echoes as a function of intensity and time (depth). By recording the intensity and time of flight (ToF) of reflected echoes from observed organs, the position of the organ can be found. To determine the velocity and motion of the organ over time, the system averages the amplitude and depth of a specific period of reflections. This mode is called motion tracking (M)-mode and is analogous to recording a video of ultrasound images (B-Mode) focused on a specific area of images. This technique is widely used in ultrasound imaging for real-time measurements of heart rate and wall thickness [34]. In this paper, the data from the proposed ultrasound system are evaluated and validated against a spirometer as a gold standard and compared to the PPG and inertial sensors as two other methods. Results indicate the superiority of our ultrasound sensor in comparison to the inertial and PPG methods under different human body motion conditions. The dataset was collected from subjects having no specific illness to perform a system validation and performance evaluation.

## 2. Methods and Principles

### 2.1. Basics of Ultrasound and Sensor Description

**Figure 1:**
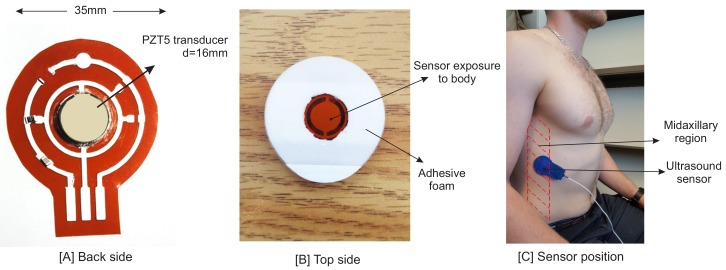
(**A**) The PZT5 piezo transducer mounted on a flexible printed PCB circuit. (**B**) The top side of the sensor. A two-sided adhesive pad is used to hold the sensor on the body. The middle round area is filled by ultrasound gel. (**C**) Sensor position in the zone of apposition (ZOA). The sensor on the back side is covered by a blue tape.

As mentioned before, the proposed system is based on the ultrasound technique. Vibration (resonant) modes of piezo-ceramic transducers depend on their shape, polarization, orientation and the direction of the electric field. The transducer used in the sensor is a 2.2 MHz PZT5 piezo disk operating in thickness mode. One main issue in piezo disk transducers operating at frequencies higher than 1 MHz is the transducer’s electrode connectivity. One side of the sensor has to be interfaced with an acoustic matching layer, with an overall thickness of λ/4 [35], while having an electrical conductivity to the circuit. λ is the acoustic wavelength in the propagation medium. Since the other side of the sensor is covered by a backing layer or left open as an air-coupled backing, the connectivity of this side is not a problem. Hence, the piezo transducer is mounted by a silver epoxy on a Kapton (polyimide) film, 125 μm in thickness. We printed a silver conductive pad (flexible silver conductive ink) on this film using a VOLTERA circuit printer to connect the transducer’s electrode to the rest of the sensor circuit. The printed substrate and finalized design are shown in Figure 1. Having silver as the main component of this epoxy, printed ink and the transducer’s electrode result in a good acoustic impedance matching between the transducer and the printed circuit layer.

To remove air gaps between the sensor and skin, a conductive soft material such as ultrasound gel is needed between the skin and the sensor. There are many alternatives, such as water, baby oil or hand cream, which have almost an equal performance. The conductive medium enables a tight bond between the skin and the sensor, enabling wave transmissions directly to the tissues underneath. One strap band or an adhesive pad is needed to hold the sensory head in its position. Moreover, the adhesive pad will ensure the sensor’s position and lessens the motion artefact of the signals.

In soft tissues, about 80% of the ultrasound wave is absorbed by the tissue, resulting in local heat production on cells [36]. Acoustic impedances and attenuation coefficients of some specific mediums and soft tissues are listed in Table 1.

Ultrasound waves are attenuated in a medium with higher value of attenuation coefficient. In the human body, bone has the highest attenuation coefficient, which hardly allows beam transmission through itself. In addition, due to the high acoustic impedance mismatch between bone and tissues, the intensity of reflections is high. Small upper body motion does not impact the ultrasound wave propagation, and these waves can still pass through the gap between the rib cage bones. Big skin movement may result in a noticeable sensor displacement and ultrasound wave blockage if the sensor faces the bones. This misplacement leads to an error in reading ultrasound reflection. Such a big displacement rarely happens when the patient is not performing intense motion.

The acoustic axial resolution of the sensor can be calculated as below by considering the average sound velocity of tissue as *c* = 1540 m/s and *f* = 2.2 MHz used in this study:

Axial resolution:

(1) $$\theta_{z}=\frac{\lambda }{2}=\frac{c}{2f}=\frac{1540}{2*2.2*10^{6}}≃0.35$$

K. M. Langen et al. [37] summarized the evaluation of the studies on diaphragm motion. The average peak-to-trough (PTT) diaphragm movement measured is 13 mm in normal breathing and 39 mm during deep breathing. Hence, the axial resolution of 0.35 mm should provide an adequate accuracy to measure the internal organ motion.

The selected piezo ceramic disc transducer has a 15 mm diameter, which is wide enough to sense reflections within the two rib bones. The ringing effect is one of the issues to consider when designing an ultrasound system. In the previous work [33,38], the transducer was operating at 1 MHz, and the piezo ringing effect period masked reflected signals from near objects. Choosing higher frequency piezo transducers not only helps to shorten the ringing effect, it also improves the axial resolution. It is worth noting that the energy of ultrasound waves attenuates as they move through tissues. The amplitude decreases approximately by 1 dB per 1 MHz per 1 cm travelled [35]. Therefore, the need to amplify the reflected signals more increases the power consumption of the circuit. Moreover, the system consumes more power in the faster switching of components. Therefore, the 2.2-MHz piezo transducer from STEMINC was selected, which optimizes our need for the ringing effect, the distance to observe objects (the diaphragm wall) and our need for power consumption.

### 2.2. Sensor Position

In this study, the goal is to investigate the motion of the diaphragm; thus, the sensor was positioned on the zone of apposition (ZOA). The ZOA is the area of the diaphragm encompassing the cylindrical portion (the part of the muscle shaped like a dome/umbrella), which corresponds to the portion directly apposed to the inner aspect of the lower rib cage [39]. The piezo sensor is placed on the right side of the body, as it is easier to see the diaphragm through the liver window [40]. The following methods were described in the literature [32,41,42,43]. The sensor was placed between Ribs 8 and 9 at the mid-axillary line (Figure 1). Having the sensor positioned in this zone would enable it to see both contraction and relaxation of the diaphragm. The diaphragm is a hyperechoic structure, which means that ultrasound can be reflected and measured [40]. Although the diaphragm is deeper than the thoracic rib cage, its motion can be seen by ultrasound from the intercostal space.

### 2.3. Study Protocol

As discussed, we are monitoring the internal organ motion, mainly the diaphragm wall. Our goal is the evaluation of the proposed system on subjects performing different body motion and position conditions. Four practical tests were done in the resting state to ensure an accurate assessment versus the gold standard. Moreover, two additional tests were applied when the subject had upper body motion to examine the system under motion artefacts. All tests were done in a sitting position. The tests are detailed as follows:

- Test-T1, normal breathing: In this test, the subject was asked to disregard the apparatus and breath normally.
- Test-T2, normal breathing with two breath holds: In this test, the subject starts with two or three normal breaths and then a long inhalation, followed by a long exhalation. This test is done to evaluate the system on breath holds, or when the subject is suffocating.
- Test-T3, fast breathing: In this test, the subject breaths fast (between 25 and 40 breathing cycles per minute) to simulate breathing after an exercise.
- Test-T4, normal and weak breathing: This test is to evaluate the capacity of the apparatus to detect weak and slow breathing. The subject is asked to be at rest and breath gently.
- Test-T5, normal breathing with hand elevation: This test is intended to evaluate the skin movements when a subject abducts the arm up to the shoulder and then completely up. As mentioned before, if the sensor is placed in front of a bone, then the measurements are impossible due to the ultrasound wave blockage.
- Test-T6, breathing with upper body motion and rotation: In this test, the subject is asked to move his/her upper body randomly in all directions. This is to evaluate the ability of the apparatus when upper body motion is occurring.

### 2.4. Ethics Approval

All participants gave their informed consent, which was approved by the appropriate ethics committee (Research Ethics Board II Office, McGill university). There are no known risks associated with the experiments asked to be performed on the subjects. In some cases, it caused dry mouth, which was eliminated by providing enough time interval between each breathing pattern and drinking water. Moreover, ultrasound has been shown on many occasions to be safe when the acoustic waves are under thresholds [44]. In a study, a similar sensor was evaluated to prove the amount of ultrasound wave intensities being less than the threshold values [38].

### 2.5. Validation Process

To validate the data obtained from the ultrasound system, a gold standard was required as a reference. Spirometer- and plethysmography-based devices are the most common clinical and commercial references. We used an SPR-BTA spirometer with GO!Link data logger software to measure oral breathing in sitting condition. A nose clip was used to prevent nasal breathing. This device measures the amount of airflow, but not the volume. Accordingly, the signal level returns to zero in breath holds. Since the proposed measurement method is a based on the volume of air the subject inhales or exhales, trapezoidal numerical integration was used to compute the approximate integral of the signal.

## 3. System Architecture

**Figure 2:**
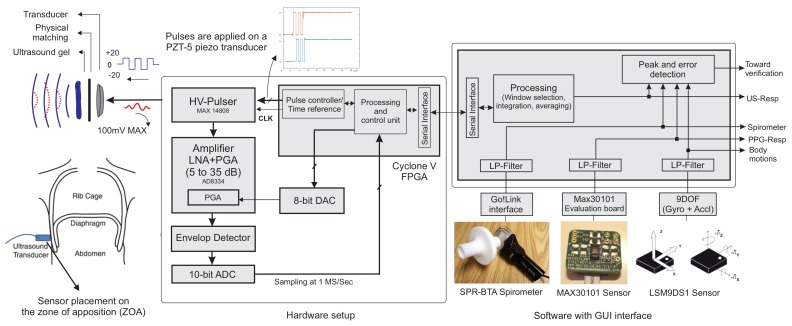
Ultrasound system designed to continuously measure and monitor respiratory cycles. The spirometer is used as a reference for ultrasound data validation and compared with PPG and inertial sensors.

Figure 2 shows the block diagram of the system designed and implemented to extract, record and monitor the ultrasound data, and validate it against the references. Digital and analogue subsystems were employed; the analogue sub-system has two main paths: a transmitter (TX) and a receiver (RX). A commercial IC MAX-14808 was deployed as the front-end high voltage (HV) pulser, switch and damper in a single chip. It hence acted as the transmitter and path separator. Furthermore, this integrated circuit (IC) is an HV pulser used to generate differential pulses up to ±20 V to increase the intensity of ultrasound waves. We employed the pulser in three main operation modes, controlled by two signals. First, the differential HV pulses were applied to the transducer, and after 5–10 pulses, the voltage returned to zero, then the damper turned on for a short time to diminish the ringing effect and possibly stored high voltage charges. In the third step, once the internal switch was connected to the RX path, the receiver circuit amplified the low voltage reflections from the sensor.

A two-stage linear amplifier with a wide passive band-pass filter magnified reflected ultrasound beams from undesired high and low frequency components of the signal. The magnified signal was passed to an envelope detector, which helped to reduce the digital signal processing work. According to the acoustic wave attenuation ratio of 1 dB per 1 MHz per 1 cm travelled in the tissue, our ultrasound wave weakened by 2.2 dB for each cm of tissue penetration. To increase the sensing of reflections from deeper tissues, the gain of the amplifier should increase linearly with the same ratio as the ultrasound waves attenuates. For this purpose, we used an analogue front-end (AFE) designed with an integrated low noise amplifier (LNA) followed by a variable gain amplifier (VGA). The gain was controlled by an analogue voltage generated by a 10-bit digital to analogue converter (DAC). Once the switch turned to RX path, the gain started increasing from 10 to almost 30 dB within 200 μs. The gain was controlled by the FPGA immediately after the switch transition. Therefore, there was no mismatch in time to generate asynchronous amplified signals. This time was chosen as it was the maximum period during which we expected to have the reflections. Finally, since the information of each echo was carried on the amplitude and time of flight of reflections, the envelope of the signal was extracted. The envelope can be produced in an analogue circuit with a simple rectifier and low pass filter or using Hilbert transform with the digitized values. The Hilbert transform method exhibited better performance, as it detected the true amplitude of the analytic signal. However, the analogue subsystem would reduce the required ADC clock and processing units dramatically since the carrier frequency was removed.

To digitize the enveloped signal, we used a 10-bit resolution analogue to digital converter (ADC) with a sampling frequency of 1 Msamples/s. It converted the analogue signal once the system switched to RX mode for 200 samples (lasting 200 μs).

A Cyclone V FPGA programmable logic device was programmed to control all blocks through digital pulses. The data were logged and processed by MATLAB (Matrix Laboratory, USA) with a user-friendly GUI interface. The processing of ultrasound data could be done completely on the FPGA. However, to make a better timing comparison between all three resources, processing was done on the computer for the purposes of our experiments.

**Figure 3:**
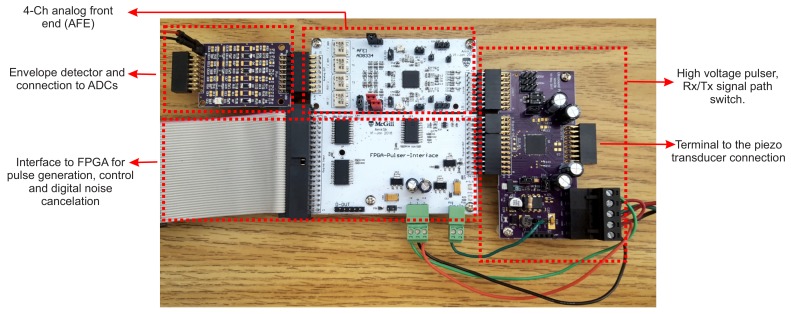
The designed 4-channel ultrasound system hardware setup consists of a high voltage pulser, an analogue front-end (AFE), an envelop detector and an ADC. It is interfaced with a Cyclone V FPGA board as a controller and data logger to the computer.

The hardware setup of the proposed system architecture is shown in Figure 3. The proposed system required +3.3-V sources for the digital and analogue integrated circuits and the differential ±5 V–±20 V from an external DC linear power supply for the transducer stimulation. It consumed 34 mA for the digital and analogue circuit. The transducer itself consumed less than 0.1 mA at different voltages. Only 393 logical blocks of a Cyclone II FPGA were used. The hardware was built using an off-the-shelf 4-channel transceiver integrated circuit (IC). The same hardware would allow further analysis of multi-sensor or different transducer configurations, as discussed in Section 5.

### 3.1. Pulse Generation and Observation

The average intensity of acoustic waves depends on the pulse repetition factor and the voltage level. As the voltage applied on the transducer increases, the intensity of acoustic waves increases, as well. As the repetition factor increases, the refreshment of this information increases, as well. In this study, we planned to measure the near tissue movements, meaning low energy acoustic pulses were sufficient to stimulate the transducer. In this study, we examined our system by pulses on differential voltages from ±5 V–±20 V applied on the transducer. Observations and measurements have shown that even a ±5-V rating is sufficient for the diaphragm motion tracking. Monitoring of subjects with thicker skin, fat and rib cages may require higher acoustic energies as the depth of penetration increases. Operation in lower voltages also consumes less energy and applies weaker ultrasound wave intensities, which might have inappreciable side-effects on the body. However, ultrasound is known for its excellent safety record.

**Figure 4:**
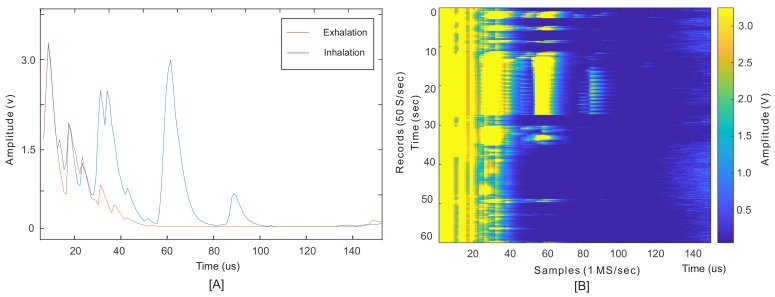
(**A**) An example of the obtained signal sampled at 1 Ms/s at the end of each inhalation and exhalation, each called a record. (**B**) One minute of records taken at 50 Hz depicts the breathing cycles.

Figure 4 shows an example of received waveforms when the sensor is placed on the ZOA. The blue and orange waveforms in Figure 4A are two examples of the envelope of echoed signals received in inhalation and exhalation, respectively. The required information lies under the peaks’ amplitude and their locations (ToF). The largest peaks marked on this figure, at times less than about 20 μs, were the result of the sensor’s ringing effect and reflections from unmatched surfaces, from sensor to skin surfaces.

Figure 4B depicts one minute of records for a subject at rest without body movements to monitor the diaphragm wall motions. These 3000 records comprise a series of envelope signals, such as the one shown in Figure 4B, recorded every 20 ms. The results were similar to M-mode ultrasound imaging, where M stands for motion tracking over time.

**Figure 5:**
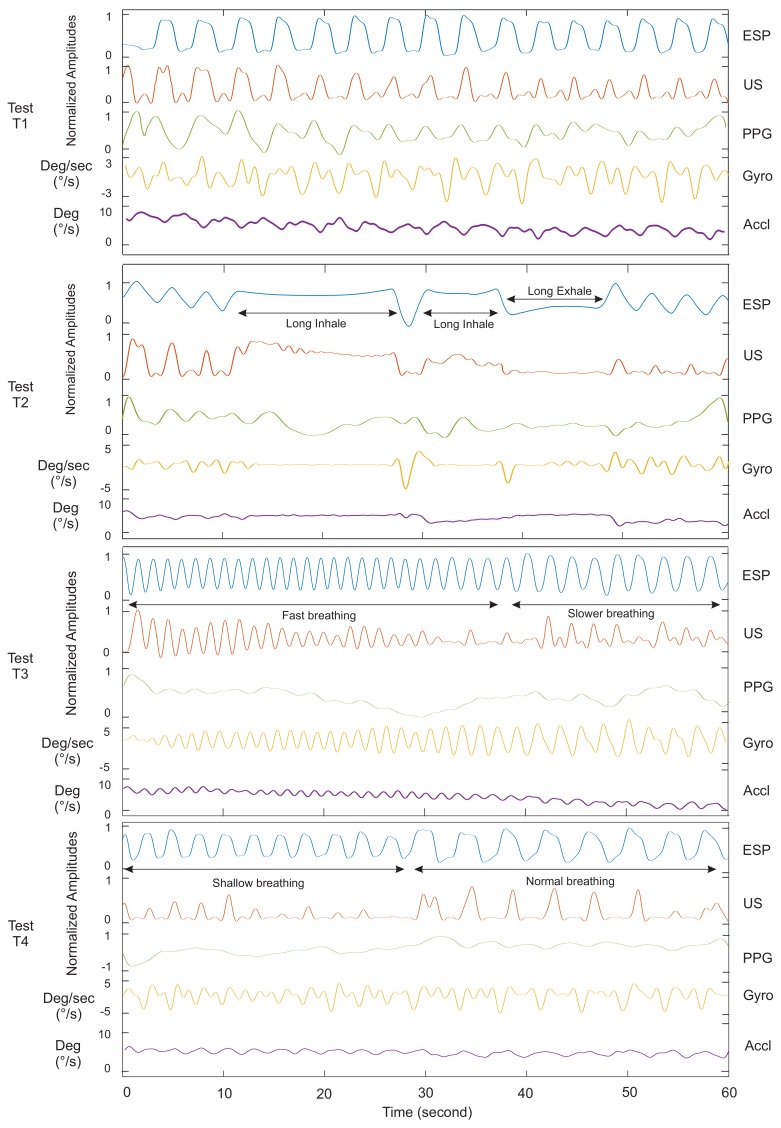
Respiratory waveforms of Subject 4 for the proposed ultrasound system (US) in comparison with the spirometer (ESP), pulse oximeter (PPG) and motion sensor (Accl/Gyro). Only four out of six types of experiments are plotted in static body conditions. All tests have close resemblance to real breathing situations.

In this test, the subject started with three normal breathing cycles. After that, two breath holds (with full inhalation and exhalation), followed by normal breathing cycles continued to the end. In this figure, the amplitude variation of each record contains the respiration information. Since these peaks were the result of an internal organ motion, rather than external motion or skin surface, the obtained information was shown to be robust to human motion. Additionally, the monitoring was based on a direct measurement of physiological human activity, and not by an indirect method. To obtain the respiratory waveform, the integral of the signal or the mean value within the desired window of each record gave a value $M_{j}$. This is the period of time the ultrasound waves were reflected from the diaphragm. Reflections before this period were the result of motion artefact, sensor ringing effect and small spikes from the internal switch when it turns into RX mode. For this study, the period was set from 22–100 μs, so ($M_{j}$) can be found as:

(2) $$M_{j}=\frac{1}{UB-LB}\sum_{i=LB}^{UB}S_{ji}$$

where *j* and *i* are the record and sample indexes, respectively (on axes *Y* and *X*). LB and UB are the lower and upper bounds of the desired window, as explained above. A series of $M_{j}$ values for all records produced the signal shown in Figure 5 as US (ultrasound).

### 3.2. Data Analysis and Peak Detection

A low-pass FIR filter was used to emit high frequency elements of the raw US signal, which was higher than 1 Hz. The same filters were used to apply to signals from the spirometer, the PPG and motion sensors. FIR filters of the same order (order 12) were used for filtering to ensure linearity in the phase of filtered data, which was an important criterion for filter selection, especially for the validation procedure. Since the respiration operation mainly changed the baseline of the PPG sensors’ data [21,24,25], the same technique was utilized in this article. We applied a band-pass filter to extract the respiratory frequency from the raw PPG signal and eliminate the extremely low frequency or DC component of the signal. The respiratory signal was a non-stationary signal, and all sensors’ data were processed in the time domain. A windowing percentile analysis [45] was performed on all low-pass filtered sensor signals to detect positive peaks. Thus, a logical output for each signal was generated containing the logic ‘1’ when the signal level passed the percentile threshold value and ‘0’ when the signal was below the threshold. The logical waveforms showing the presence of each detected inhalation and exhalation in all signals were utilized in the statistical evaluations in Table 2. The percentile value was adaptive to the mean value of the signal in current and past windows.

## 4. Results

For all the listed tests, the inertial sensor was placed on the ultrasound sensor to monitor the body motion and motions due to breathing. This sensor provided three-axis accelerometer and gyroscope data at the same rate as ultrasound. We used the MAX30101 multi-sensory board, which provides a proven design to evaluate the integrated pulse-oximetry and heart rate monitoring. PPG sensor was selected as a comparative technique to the proposed ultrasound system. Some studies proposed the use of PPG sensors for respiration estimation as discussed in the Introduction. However, to our best knowledge, there is no evident practical test of respiratory monitoring when the breathing is fast or the subject is moving. In this study, we evaluated the PPG under the same conditions as our proposed system.

**Figure 6:**
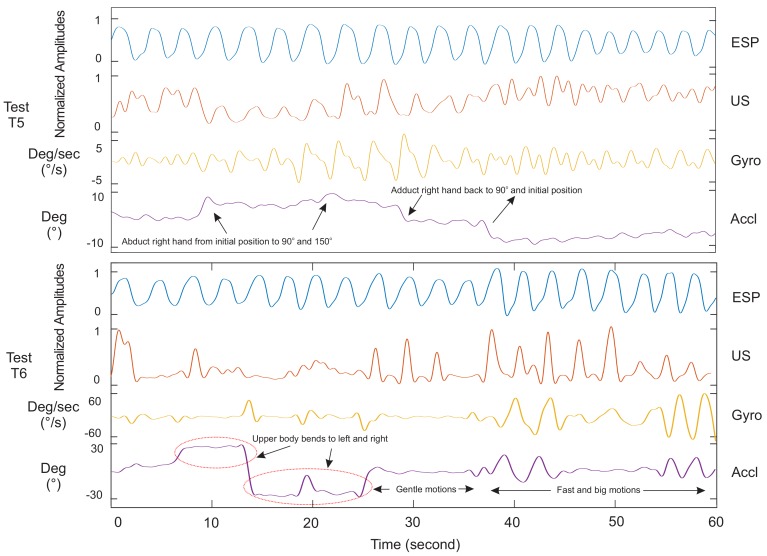
Respiratory waveforms of Subject 4 for the proposed ultrasound system (US) in comparison with the spirometer (ESP) and motion sensor (Accl/Gyro) under human body motion condition. Since the PPG data were found to be sensitive to the motions, data collection was skipped for analysis in T5 and T6.

Figure 5 and Figure 6 show the signals of our ultrasound system (US), PPG, spirometer and motion sensors for a minute record, measured on Subject 4. It helps to compare waveforms visually and have a better assessment of the proposed system functionalities. Tests T1, T2, T3 and T4 in Figure 5 were experiments without body motions, and Tests T5–T6 in Figure 6 were designed to evaluate the system with body motion. Our observations and analysis of the PPG data when the subject was moving showed uncertainty in the respiratory waveforms. Therefore, we skipped showing the PPG data for Tests T5 and T6 in Figure 6. The accelerometer and gyroscope data in this figure show the seating position and motion of the body.

**Figure A1:**
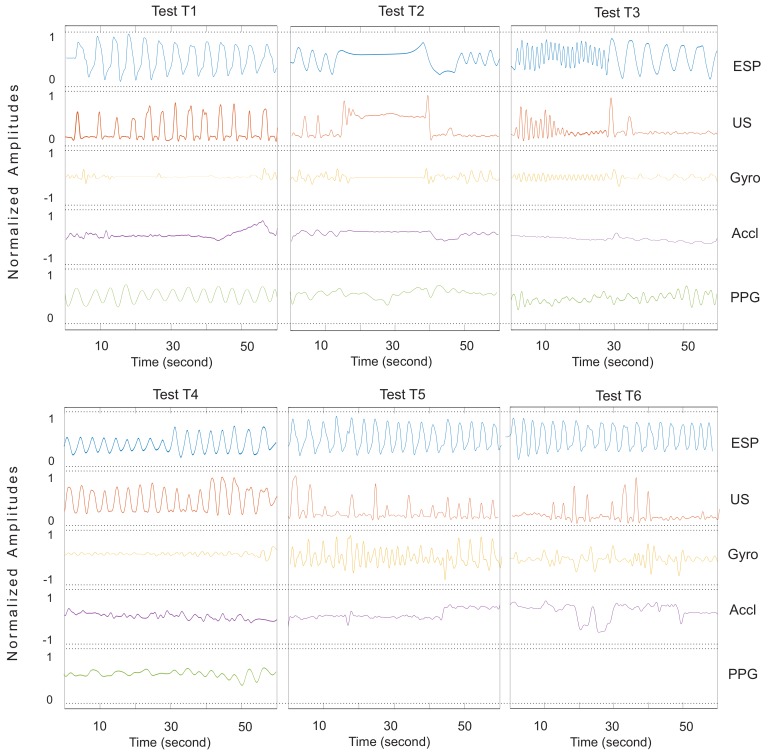
Respiratory waveforms of Subject 1.

**Figure A2:**
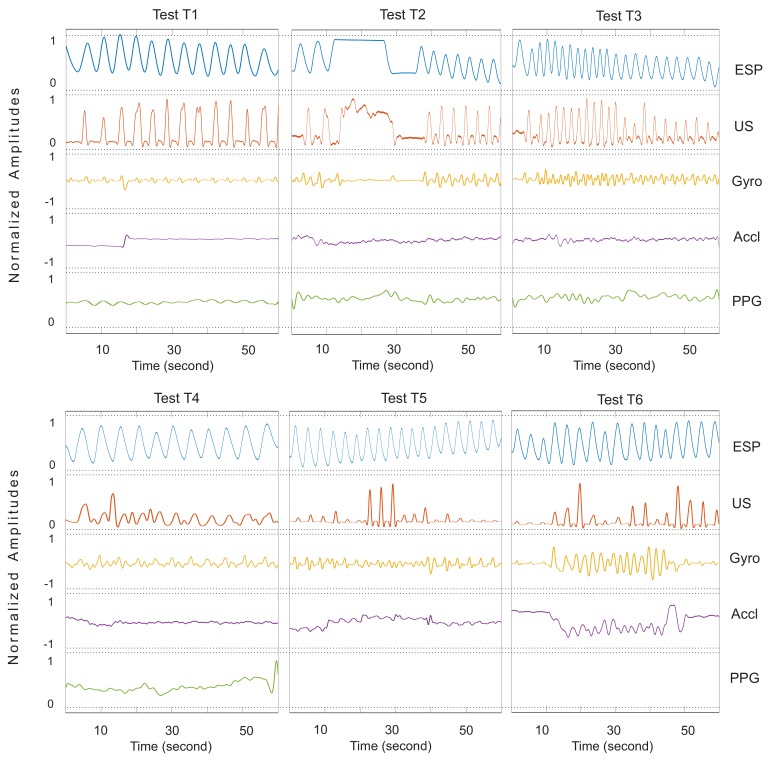
Respiratory waveforms of Subject 2.

**Figure A3:**
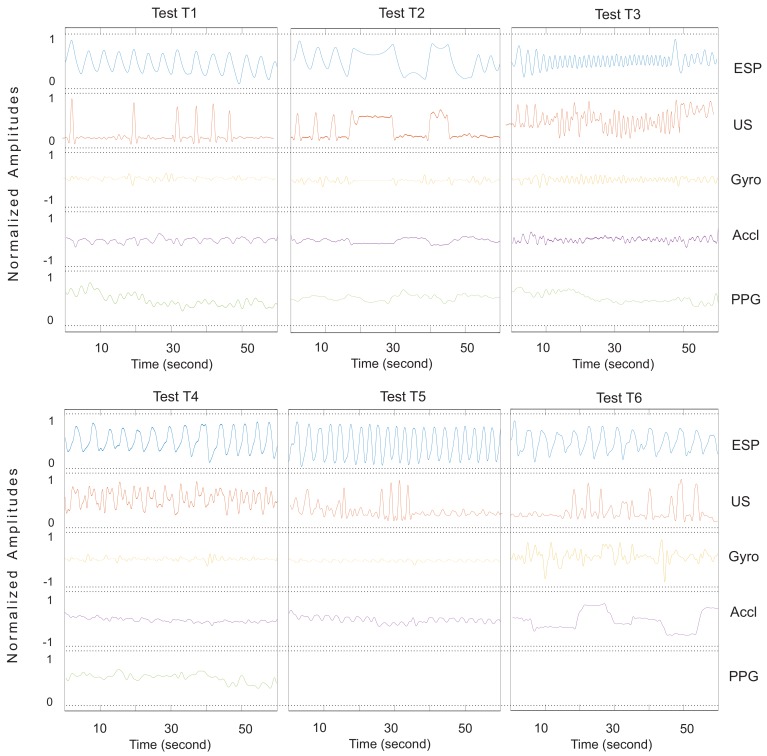
Respiratory waveforms of Subject 3.

**Figure A4:**
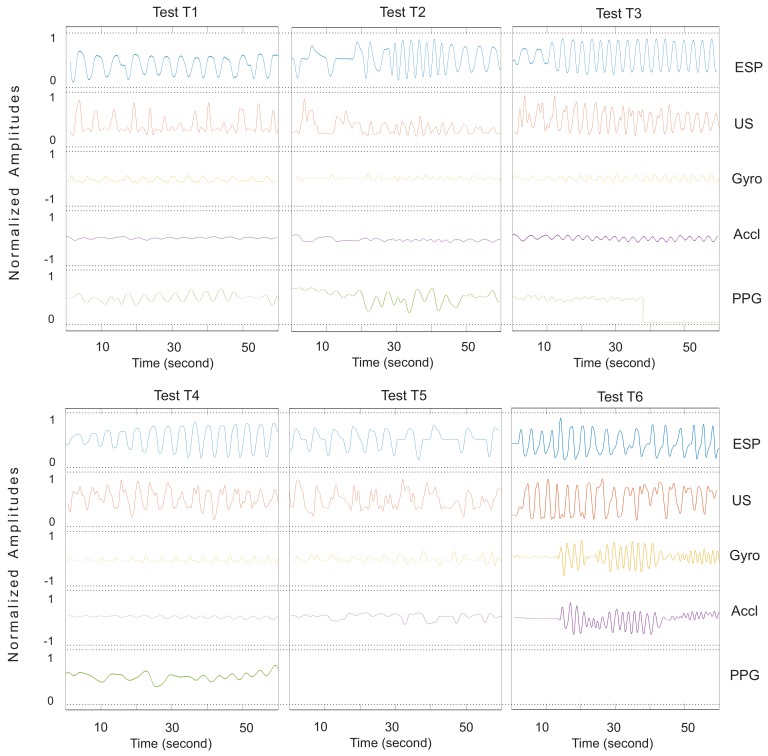
Respiratory waveforms of Subject 5. The PPG sensor was detached from the subject’s finger in the last 15 s.

**Figure A5:**
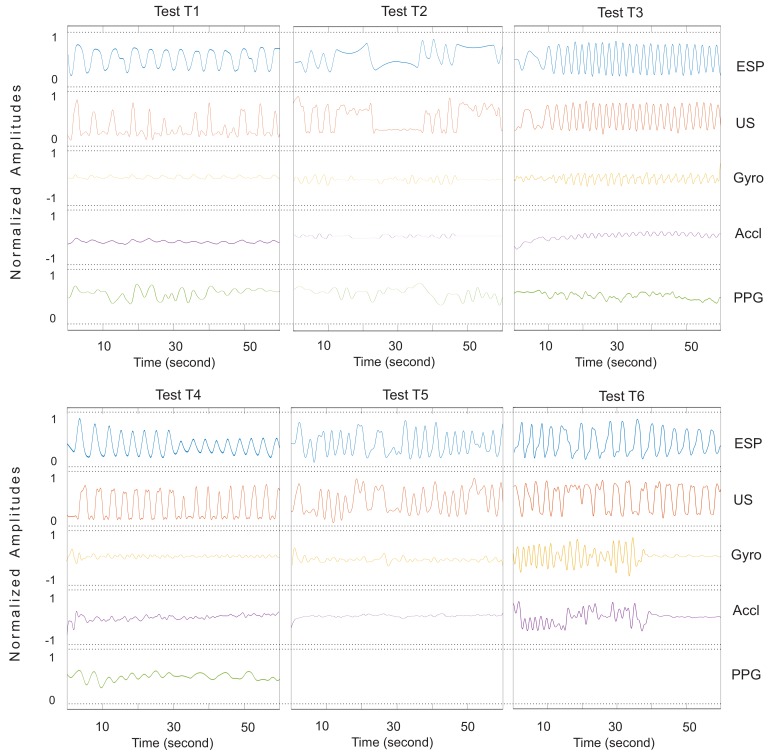
Respiratory waveforms of Subject 6.

Among the gyroscope and accelerometer sensors data, the gyroscope was found to produce a better signal. Although the magnitude of data from these two sensors was extremely small, according to our data, the respiratory waveform from the gyroscope was still more detectable than the accelerometer. Figure 5 and Figure A1, Figure A2, Figure A3, Figure A4 and Figure A5 clearly show the difference.

### 4.1. Ultrasound Data Validation: Static Body Posture

Test T1 in Figure 5 consisted of 16 normal inhalations and exhalations per minute. As evident in this figure, the ultrasound system (US) had a clear breathing detection versus the reference (ESP). The PPG sensor’s data followed the respiratory waveform with a lower accuracy and intensity, while the gyroscope data had peaks that could be counted as wrong detections.

Test T2 consisted of normal breathing with two breath holds. All subjects began with three normal breaths. Then, they were asked to hold their inhaled breaths for 10–20 s followed by a complete exhalation. The test continued with normal breathing to the end after the breath holds. This test was done to simulate an apnoea situation and the feasibility of an apnoea detection using the proposed respiratory monitoring. According to the signals of this test in Figure 5, the PPG sensor was unable to follow the overall breathing patterns except three true detections at the beginning. Moreover, peak signals generated by this sensor could be realized as breathing activities. More signals in the Appendix confirm this fact. The inertial sensor could provide a better signal than the PPG; however, the sensor generated some peaks at the end, which could be counted as breathing. In addition to the breathing detection, the system was able to show the diaphragm efforts when applying pressure to open the airway. In a study by Holland et al. [46], they showed that breath holding did not eliminate the motion of the diaphragm, and the diaphragm moved upward during a breath hold with a constant velocity of 0.15 mm/s. This phenomenon was evident in our data shown in the Figure 5 US of Test T2. The mean amplitude of the signal lowered almost constantly by time.

**Figure 7:**
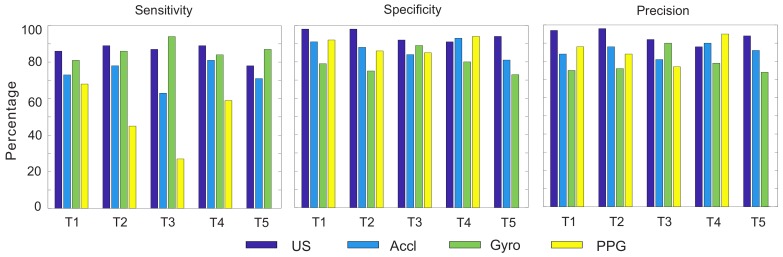
Graphical presentation of the values listed in Table 2.

Test T3 was the same as Test T1, except that the breathing was faster. This test was done not only to evaluate the performance of the proposed system, but to compare against the PPG response to fast breathing. Our analysis showed a high correlation and breath detection of the ultrasound sensor versus the gold standard. However, four breaths were not detected by the ultrasound system, which was only 11% of the total breathing cycles in this test. Inertial sensors performed better than the PPG sensor in this test. Detailed results are listed in Table 2 and Figure 7.

Test T4 was similar to T1 except in comparing how the ultrasound sensor responded to weak and normal breathings versus the reference. The subject began with shallow breathing for almost 30 s and continued with normal breathing to the end. Our US signal could detect the weak breathing, but the amplitude of the signal was low and failed at three points. Note that some subjects had very weak breathing, which was the reason breath detection was missing in Figure 5T4. During the same test, the PPG sensor did not provide any relevant signal regarding the weak breathing, and positive peak times in the Gyro signal were not distinguishable from negative peaks. All sensors operated well in normal breathing for the second half of the test. It is worth noting that the US system rarely generated false readings.

### 4.2. Ultrasound Data Validation: Dynamic Body Posture

In this section, the proposed system is assessed versus other methods when subjects had arbitrary upper body motions. Results are shown in Figure 6. We performed Test T5 to evaluate if the US sensor moved with hand elevation and its consequence on respiratory data. In this test, the subject started with two normal breaths with the arm relaxed on each side. Then, he/she abducted his/her right arm up to 90 degrees for two more breaths in that position, followed by an abduction of his/her arm up to almost 150 degrees and breathing two times again. Finally, he/she continued breathing while bringing the hand back to 90 degrees and then the initial position. The right hand was chosen because the sensor was placed on the right side of the body. The skin around the ZOA shifted vertically following hand elevation. This shift caused the sensor to be displaced in front of a rib cage bone. Our analysis of subjects showed that the system operated ideally as long the elevation was up to 90 degrees. The system failed to detect respiration a few times when the hand was elevated more than 90 degrees.

Finally, in Test T6, the proposed method was examined against upper body motion. An example of this test is shown in Figure 6 while the subject bent to left and right for 25 s. Then, the subject was asked to do slower or more gentle motions for 10 s. The subject continued to the end with fast and big motions while breathing normally. The reported results and observations showed a fairly correlated data versus the gold standard when the subject’s body had motions. Although the sensor displacement on the first 25 s did not allow the system to track the diaphragm’s movements (the similar issue in Test T5), the system showed better performance to the rest of the tests, as seen either in Figure 6 or other reported data in the Appendix. Due to the hand and body motions, the PPG data collection was skipped in these two tests as respiratory waveforms of these sensors were susceptible to motion artefacts [26].

Table 2 is a list of the sensitivity, specificity and precision values of all methods with regard to the reference and plotted in Figure 7 for an easier distinction. The sensitivity or true positive rate (TPR) was calculated by the equation below:

(3) $$Sensitivity\left(TPR\right)=\frac{TP}{TP+FN}$$

In this equation, the true positive (TP) was the number of correctly-detected full breathing operations and false negative (FN) was the number of breaths wrongly classified as negative. In this table, the total number of breaths per minute of the spirometer is listed. True negative (TN) was considered positive when both the gold standard (spirometer) and the sensor did not detect any breathing. For example, if the spirometer and the sensor did not record any breathing when no breathing was occurring in between two breathing cycle or when the subject was holding their breath, it was considered a true negative. The specificity or the true negative rate was calculated to identify the proportion of non-breaths that were correctly identified. It could be calculated using the equation below:

(4) $$Specificity\left(SPC\right)=\frac{TN}{TN+FP}$$

In addition to sensitivity, the precision or positive predictive value (PPV) was calculated by Equation (5). In this equation, the false positive (FP) is the total number of breathing operations detected by an error. This error in the ultrasound system could be a result of sensor displacement, which rarely happened during our experiments. This value was noticeably high for the gyroscope. The statistics of Test T6 were not calculated for the gyroscope, accelerometer and PPG sensors due to the extremely noisy and unusable data.

(5) $$Precision\left(PPV\right)=\frac{TP}{TP+FP}$$

According to the above-mentioned results and visual observations, the authors did not find a relationship between the body specification (Table 3) and system performance.

### 4.3. Non-Respiratory Movements

As mentioned before regarding the desired window, the system looked into reflected ultrasound waves from internal organs, mainly the diaphragm. Among all the subject tests of T6 in this article, none of the tests showed any correlation with body motion. In some tests where the subject held the breath for a longer time, there were some small peaks in the signal due to the heart operation. These spikes were visible in most of the T2 tests of this article during the breath holds. Heart contractions transmitted motions to the surrounding organs, such as the pericardium sac and the diaphragm [47].

## 5. Discussion

In reference to the provided data, the proposed ultrasound system provides a stable respiratory signal using only one sensor placed in the zone of apposition. Although the system is sensitive to large sensor displacements, the respiratory signal could be monitored even in gentle body motions, at different breathing rates and intensities. In addition, it does not generate false detection in comparison to the other employed methods. Based on observations of all experiments, a weaker correlation is found between the PPG, Accl and Gyro versus the spirometer as the gold standard in the T1 to T4 test methods. As per the discussion on indirect methods for respiratory monitoring systems in the Introduction, the PPG sensor responds slower to the respiratory trend and is non-responsive when the sensor or body is not physically stable. Respiratory-induced motions on the body are negligible compared to the human motions during daily activities. This is while the respiratory signal is detectable by the proposed ultrasound system even when the body is moving, as presented in all T6 tests in Figure 6 and the Appendix figures.

To improve the displacement sensitivity, an array of transducers will enable the system to track the diaphragm motion reliably in the future. In this way, at least one transducer will trace motion changes of the diaphragm even if others are blocked by the ribs. An array of three transducers placed side by side within the intercostal space could be a solution to overcome this issue. Note that transducers placed on a flexible material will not cause any discomfort, nor inconvenience for the subjects.

Our tests show that when the subjects were performing long oral breathing test with the spirometer, they felt exhausted and dizzy when the test reached a 50–60-s period. Further analysis is scheduled to apply prolonged tests based on a different reference. The system uses a bulky setup with one wired connection to the sensor. In future, integrated wireless hardware will be developed to replace the wire connectivity and bulky evaluation setup. System evaluations of the noninvasive ventilated (NIV) [48] subjects will be addressed.

## 6. Conclusions

In this paper, we presented a direct respiratory monitoring system based on the pulsed ultrasound technique. The sensor consists of a PZT5 piezo transducer mounted on a flexible surface in direct-contact with the skin surface. The sensor should be placed in the zone of apposition (ZOA) on the right side of the body to observe the motion of the diaphragm. The proposed diaphragm motion tracking sensor provides fairly good detection of respiratory cycles in different breathing patterns. The system was compared against a spirometer as the gold standard. In addition, we compared our measurements with inertial and photoplethysmography (PPG) sensors. Our ultrasound sensor was less affected by upper body motions in comparison to the inertial and PPG sensors.

## Figures and Tables

**Table 1:** Acoustic properties of biological tissues.

Medium	Attenuation Coefficient (dB/cm)	Acoustic Impedance (kg/m^2^·s or rayl) $*10^{6}$
Water	0.002	1.48
Blood	0.18	1.61
Fat	0.63	1.38
Muscle	1.3–3.3	1.62
Bone	5.0	6.0
Silver	16	5.14
Ultrasound Gel	-	1.6

**Table 2:** Statistical measurements of all experimental tests on 6 subjects. Results are compared with the spirometer as a reference. The average number of breaths per minute of all tests are measured by spirometer. As shown in Figure 5, T5 and T6, the inertial and PPG sensors are not applicable (N/A) to respiratory monitoring when the subject’s body moves.

Test Number	Breaths per Minute	Sensitivity (%)				Specificity (%)				Precision (%)			
		US	Accl	Gyro	PPG	US	Accl	Gyro	PPG	US	Accl	Gyro	PPG
Test 1	13	86	73	81	68	98	91	79	92	98	84	75	88
Test 2	11	89	78	86	48	98	88	75	86	98	88	76	84
Test 3	25	87	63	94	27	92	84	90	85	92	81	90	77
Test 4	16	89	81	84	59	91	93	80	94	88	90	79	95
Test 5	16	78	71	87	N/A	94	81	73	N/A	94	86	74	N/A
Test 6	17	73	N/A	N/A	N/A	89	N/A	N/A	N/A	89	N/A	N/A	N/A
Average	N/A	84	73	86	51	93	86	79	86	94	87	79	89

**Table 3:** Summary of subjects’ specifications. CC is the chest circumference.

ID	Age (year)	Height (cm)	Weight (kg)	CC (cm)
1	36	173	73	84
2	29	176	65	81
3	26	175	75	84
4	25	183	95	88
5	27	165	50	74
6	34	175	70	74

## Appendix A

Respiratory waveforms of the other five subjects (Subjects 1, 2, 3, 5 and 6) are plotted here. The proposed ultrasound system (US) is compared with the spirometer (ESP) as our reference, pulse oximeter (PPG) and motion sensors (Accl and Gyro). AS mentioned before in Section 4, since the PPG sensor is sensitive to the motions of the sensor and human body, the PPG data collection is skipped for Tests T5 and T6 of the following figures. Note that the Gyro and Accl signals are not necessarily synchronous with the breathing rates in the T5 and T6 tests. For the convenience of the readers, the amplitude of all signals was normalized to make the visual comparison easier. It is worth noting that the amplitude variations of the Gyro and Accl signals in the T5 and T6 tests were almost ten-times greater than the other four tests.

## References

[1] Vivier E., Dessap A.M., Dimassi S., Vargas F., Lyazidi A., Thille A.W., Brochard L. (2012). Diaphragm ultrasonography to estimate the work of breathing during non-invasive ventilation. Intensive Care Med..

[2] Sigala I., Vassilakopoulos T. (2017). Diaphragmatic ultrasound as a monitoring tool in the intensive care unit. Ann. Transl. Med..

[3] Laghi F., Tobin M.J. (2003). Disorders of the respiratory muscles. Am. J. Respir. Crit. Care Med..

[4] Khan A., Morgenthaler T.I., Ramar K. (2014). Sleep disordered breathing in isolated unilateral and bilateral diaphragmatic dysfunction. J. Clin. Sleep Med. JCSM Off. Publ. Am. Acad. Sleep Med..

[5] Heldt G.P., Ward R.J. (2016). Evaluation of Ultrasound-Based Sensor to Monitor Respiratory and Nonrespiratory Movement and Timing in Infants. IEEE Trans. Biomed. Eng..

[6] Umbrello M., Formenti P., Longhi D., Galimberti A., Piva I., Pezzi A., Mistraletti G., Marini J.J., Iapichino G. (2015). Diaphragm ultrasound as indicator of respiratory effort in critically ill patients undergoing assisted mechanical ventilation: A pilot clinical study. Crit. Care.

[7] Javaheri S., Dempsey J. (2013). Central sleep apnoea. Comp. Physiol..

[8] Sinharay A., Rakshit R., Khasnobish A., Chakravarty T., Ghosh D., Pal A. (2017). The Ultrasonic Directional Tidal Breathing Pattern Sensor: Equitable Design Realization Based on Phase Information. Sensors.

[9] Araujo G., Freire R., Silva J., Oliveira A., Jaguaribe E. Breathing flow measurement with constant temperature hot-wire anemometer for forced oscillations technique. Proceedings of the 21st IEEE Instrumentation and Measurement Technology Conference (IEEE Cat. No.04CH37510).

[10] Bai Y.W., Li W.T., Chen Y.W. Design and implementation of an embedded monitor system for detection of a patient’s breath by double webcams. Proceedings of the 12th IEEE International Conference on e-Health Networking, Applications and Services.

[11] Bu N., Ueno N., Fukuda O. Monitoring of Respiration and Heartbeat during Sleep using a Flexible Piezoelectric Film Sensor and Empirical Mode Decomposition. Proceedings of the 29th Annual International Conference of the IEEE Engineering in Medicine and Biology Society.

[12] Loriga G., Taccini N., Rossi D.D., Paradiso R. Textile Sensing Interfaces for Cardiopulmonary Signs Monitoring. Proceedings of the 2005 IEEE Engineering in Medicine and Biology 27th Annual Conference.

[13] Krehel M., Schmid M., Rossi R.M., Boesel L.F., Bona G.L., Scherer L.J. (2014). An optical fibre-based sensor for respiratory monitoring. Sensors.

[14] Massaroni C., Saccomandi P., Schena E. (2015). Medical smart textiles based on fibre optic technology: An overview. J. Funct. Biomater..

[15] Jeong J., Jang Y., Lee I., Shin S., Kim S. Wearable respiratory rate monitoring using piezo-resistive fabric sensor. Proceedings of the World Congress on Medical Physics and Biomedical Engineering.

[16] Fekr A.R., Radecka K., Zilic Z. (2015). Design and evaluation of an intelligent remote tidal volume variability monitoring system in e-health applications. IEEE J. Biomed. Health Inf..

[17] Arlotto P., Grimaldi M., Naeck R., Ginoux J.M. (2014). An Ultrasonic Contactless Sensor for Breathing Monitoring. Sensors.

[18] Fouzas S., Priftis K.N., Anthracopoulos M.B. (2011). Pulse oximetry in pediatric practice. Pediatrics.

[19] Sahni R. (2012). Noninvasive monitoring by photoplethysmography. Clinics Perinatol..

[20] Landsverk S.A., Hoiseth L.O., Kvandal P., Hisdal J., Skare O., Kirkeboen K.A. (2008). Poor agreement between respiratory variations in pulse oximetry photoplethysmographic waveform amplitude and pulse pressure in intensive care unit patients. J. Am. Soc. Anesthesiol..

[21] Charlton P.H., Bonnici T., Tarassenko L., Alastruey J., Clifton D.A., Beale R., Watkinson P.J. (2017). Extraction of respiratory signals from the electrocardiogram and photoplethysmogram: Technical and physiological determinants. Physiol. Meas..

[22] Shelley K.H., Jablonka D.H., Awad A.A., Stout R.G., Rezkanna H., Silverman D.G. (2006). What is the best site for measuring the effect of ventilation on the pulse oximeter waveform?. Anesthesia Analgesia.

[23] Iyriboz Y., Powers S., Morrow J., Ayers D., Landry G. (1991). Accuracy of pulse oximeters in estimating heart rate at rest and during exercise. Br. J. Sports Med..

[24] Charlton P., Birrenkott D.A., Bonnici T., Pimentel M.A., Johnson A.E., Alastruey J., Tarassenko L., Watkinson P.J., Beale R., Clifton D.A. (2017). Breathing rate estimation from the electrocardiogram and photoplethysmogram: A review. IEEE Rev. Biomed. Eng..

[25] Meredith D., Clifton D., Charlton P., Brooks J., Pugh C., Tarassenko L. (2012). Photoplethysmographic derivation of respiratory rate: A review of relevant physiology. J. Med. Eng. Technol..

[26] Brochard L., Martin G.S., Blanch L., Pelosi P., Belda F.J., Jubran A., Gattinoni L., Mancebo J., Ranieri V.M., Richard J.C.M. (2012). Clinical review: Respiratory monitoring in the ICU-a consensus of 16. Crit. Care.

[27] Felblinger J., Boesch C. (1997). Amplitude demodulation of the electrocardiogram signal (ECG) for respiration monitoring and compensation during MR examinations. Magn. Reson. Med..

[28] Moody G.B., Mark R.G., Zoccola A., Mantero S. (1985). Derivation of respiratory signals from multi-lead ECGs. Comput. Cardiol..

[29] Wang F.T., Chan H.L., Wang C.L., Jian H.M., Lin S.H. (2015). Instantaneous respiratory estimation from thoracic impedance by empirical mode decomposition. Sensors.

[30] Pantelopoulos A., Bourbakis N.G. (2010). A Survey on Wearable Sensor-Based Systems for Health Monitoring and Prognosis. IEEE Trans. Syst. Man Cybern. Part C Appl. Rev..

[31] Sweeney D.A. (2015). Point-of-Care Ultrasound. Crit. Care Med..

[32] Zambon M., Beccaria P., Matsuno J., Gemma M., Frati E., Colombo S., Cabrini L., Landoni G., Zangrillo A. (2016). Mechanical ventilation and diaphragmatic atrophy in critically ill patients: An ultrasound study. Crit. Care Med..

[33] Shahshahani A., Bhadra S., Zilic Z. A Continuous Respiratory Monitoring System Using Ultrasound Piezo Transducer. Proceedings of the 2018 IEEE International Symposium on Circuits and Systems (ISCAS).

[34] Garcia M.J., Rodriguez L., Ares M., Griffin B.P., Klein A.L., Stewart W.J., Thomas J.D. (1996). Myocardial wall velocity assessment by pulsed Doppler tissue imaging: Characteristic findings in normal subjects. Am. Heart J..

[35] Tole N.M. (2005). Basic Physics of Ultrasonic Imaging.

[36] Shung K. (1998). Diagnostic Ultrasound: Imaging and Blood Flow Measurements.

[37] Langen K., Jones D. (2001). Organ motion and its management. Int. J. Radiat. Oncol. Biol. Phys..

[38] Shahshahani A., Nafchi D.R., Zilic Z. Ultrasound sensors and its application in human heart rate monitoring. Proceedings of the 2017 IEEE International Symposium on Circuits and Systems (ISCAS).

[39] De A.T., Estenne M. (1988). Functional anatomy of the respiratory muscles. Clin. Chest Med..

[40] Sarwal A., Walker F.O., Cartwright M.S. (2013). Neuromuscular ultrasound for evaluation of the diaphragm. Muscle Nerve.

[41] McKenzie D., Gandevia S., Gorman R., Southon F. (1994). Dynamic changes in the zone of apposition and diaphragm length during maximal respiratory efforts. Thorax.

[42] Wait J.L., Nahormek P.A., Yost W.T., Rochester D.P. (1989). Diaphragmatic thickness-lung volume relationship in vivo. J. Appl. Physiol..

[43] Zambon M., Cabrini L., Zangrillo A. (2013). Diaphragmatic ultrasound in critically ill patients. Annual Update in Intensive Care and Emergency Medicine.

[44] (2008). Guidance for Industry and FDA Staff Information for Manufacturers Seeking Marketing Clearance of Diagnostic Ultrasound Systems and Transducers.

[45] OMSignal Validation of Breathing Rate Algorithm During Running.

[46] Holland A.E., Goldfarb J.W., Edelman R.R. (1998). Diaphragmatic and cardiac motion during suspended breathing: preliminary experience and implications for breath-hold MR imaging. Radiology.

[47] Scott A.D., Keegan J., Firmin D.N. (2009). Motion in cardiovascular MR imaging. Radiology.

[48] Caironi G., Gadda G., Rossi R., Bellone A., Esquinas A.M. (2016). Monitoring Patients During Noninvasive Ventilation: The Clinical Point of View. Noninvasive Mechanical Ventilation: Theory, Equipment, and Clinical Applications.

